# Immune-Modulation by the Human Respiratory Syncytial Virus: Focus on Dendritic Cells

**DOI:** 10.3389/fimmu.2019.00810

**Published:** 2019-04-15

**Authors:** Eduardo I. Tognarelli, Susan M. Bueno, Pablo A. González

**Affiliations:** Millennium Institute on Immunology and Immunotherapy, Departamento de Genética Molecular y Microbiología, Facultad de Ciencias Biológicas, Pontificia Universidad Católica de Chile, Santiago, Chile

**Keywords:** dendritic cells (DCs), DC maturation, antigen presentation, T cell activation, inflammation, recurrent infection, immune evasion, immunity

## Abstract

The human respiratory syncytial virus (hRSV) is the leading cause of pneumonia in infants and produces a significant burden in the elderly. It can also infect and produce disease in otherwise healthy adults and recurrently infect those previously exposed to the virus. Importantly, recurrent infections are not necessarily a consequence of antigenic variability, as described for other respiratory viruses, but most likely due to the capacity of this virus to interfere with the host's immune response and the establishment of a protective and long-lasting immunity. Although some genes encoded by hRSV are known to have a direct participation in immune evasion, it seems that repeated infection is mainly given by its capacity to modulate immune components in such a way to promote non-optimal antiviral responses in the host. Importantly, hRSV is known to interfere with dendritic cell (DC) function, which are key cells involved in establishing and regulating protective virus-specific immunity. Notably, hRSV infects DCs, alters their maturation, migration to lymph nodes and their capacity to activate virus-specific T cells, which likely impacts the host antiviral response against this virus. Here, we review and discuss the most important and recent findings related to DC modulation by hRSV, which might be at the basis of recurrent infections in previously infected individuals and hRSV-induced disease. A focus on the interaction between DCs and hRSV will likely contribute to the development of effective prophylactic and antiviral strategies against this virus.

## Introduction

The human respiratory syncytial virus (hRSV) is the leading cause of infant pneumonia worldwide and also elicits significant morbidity in the elderly and children (1–3). Importantly, infants with partial airway development due to premature birth, airway hyperreactivity, pulmonary hypertension, cystic fibrosis, Down syndrome, neurologic conditions, congenital heart disease, and those that are immunosuppressed are at increased risk of developing severe complications due to hRSV infection, which may even lead to death (4, 5). Nevertheless, individuals that are otherwise healthy, such as infants 2 months old or older can also be infected with hRSV and suffer respiratory illness leading to significant morbidity and eventually life-threatening disease, mainly because of complicated pneumonia (3, 6, 7). Noteworthy, at present there are no vaccines available against hRSV, yet many are under development and being assessed clinically, although few would be suitable for direct application on to newborns (8–11).

An important feature of hRSV is that it is capable of re-infecting healthy children and adults that have been previously infected with this virus (12, 13). hRSV can be classified into two groups (A or B) that mainly differentiate from each other based on nucleotide variability in the attachment glycoprotein G (14). Thanks to affordable sequencing costs and high-throughput sequencing techniques, at present circulating hRSV isolates, can be further sub-classified into at least 14 A genotype groups and 23 B genotypes groups, based on similarities of the G protein gene (14). Furthermore, improved access to whole genome sequencing has opened the possibility for molecular classification and molecular epidemiology studies on hRSV (15). However, despite nucleotide and antigenic variability in the attachment G protein of hRSV, recurrent infections with the same virus can occur at a high frequency within healthy individuals. For instance, adults that had a previous natural infection with hRSV and were then exposed to a virus belonging to the same strain group subsequently manifested several reinfections. At 26 months, 73% of individuals were shown to have two or more re-infections, and 47% had three or more infections (16). Thus, other immune-evasion mechanisms distinct from antigenic variation are likely at the base of host reinfections with hRSV. Because relatively few hRSV-encoded genes are known to directly interfere with the host's antiviral response in a somewhat direct manner, one could suggest that the capacity of hRSV to repeatedly infect the host may derive from its ability to elicit an adaptive antiviral immune response that is non-optimal for the host. Indeed, hRSV has been exhaustively described to skew the host's antiviral immune response toward phenotypes that promote exacerbated lung inflammation in response to lung infection which favor the virus (17–20). Importantly, hRSV lung infection can induce macrophages and monocyte-derived macrophages in this tissue to upregulate the surface expression of PD-L1, which will likely have adverse effects over the function of T cells (21). Furthermore, hRSV can elicit human neonatal regulatory B cells to secrete IL-10, which may also result in non-optimal antiviral T cell responses (22). However, an immune cell of choice targeted by hRSV seems to be dendritic cells (DCs), critical immune cells that initiate and regulate antigen-specific adaptive antiviral immunes responses. Indeed, the phenotype and function of these cells have been broadly reported to be modulated during hRSV infection, both *in vitro* and *in vivo*. Here, we review the latest studies that describe the interaction between hRSV and DCs and how the outcome affects relevant functions of these cells, which will ultimately impact the establishment of an effective antiviral response against hRSV in the host.

## hRSV Genes and the Virion Structure

**Figure 1:**
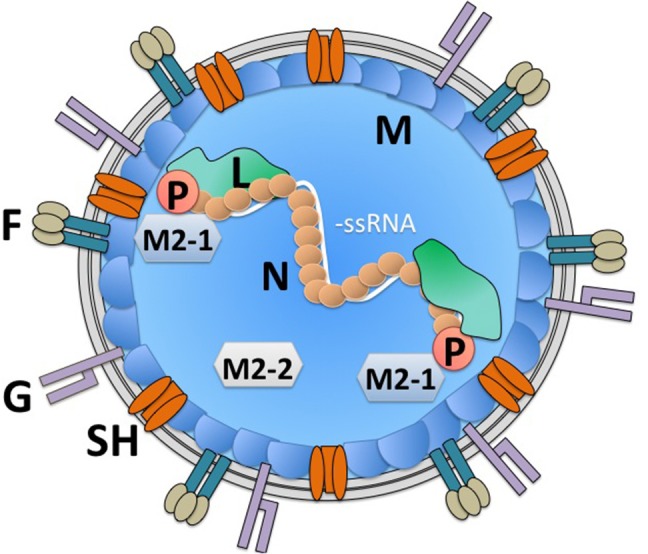
hRSV virion structure. The hRSV genome is a negative-sense single-stranded RNA virus. Its genome is wrapped by the nucleoprotein (N). An RNA-dependent RNA polymerase (L) is associated to the viral genome in the virion. Also, within the virion is the viral phosphoprotein (P), which is required by the L polymerase for its function. Additionally, within the virion are the viral proteins M2-1 and M2-2, derived from a single mRNA, which modulate transcription of viral genes and genome replication by the L polymerase. Beneath the virus envelope is the matrix protein (M), which has been described to inhibit host gene translation in the nucleus of infected cells. Three proteins are immersed in the virus envelope: the small hydrophobic (SH) protein, which forms a viroporin, that transports cations, the attachment glycoprotein (G) and the fusion protein, which arranges as a trimer on the virion surface (F). The viral proteins NS1 and NS2 are non-structural and hence, are not found within the virion.

hRSV is an enveloped, negative-sense, and single-stranded RNA virus that encodes 10 genes that are translated into 11 proteins (Figure 1) (23). Its replication and gene transcription occur in the cytoplasm, thanks to the aid of an RNA-dependent RNA-polymerase that is encoded within the viral genome by the L gene (24, 25). For its adequate function, the L protein requires the viral phosphoprotein P, which associates to this RNA polymerase (26, 27). Importantly, the replication of the viral genome and the transcription of its genes are modulated by the hRSV-encoded factors M2-1 and M2-2, which are generated from a common mRNA transcript by a ribosome shift that occurs on the mRNA after producing the M2-1 protein; initiation of M2-2 takes place at a start codon that overlaps with the M2-1 open reading frame (ORF) (23, 28). Noteworthy, in the virion and infected cells, the viral genome is covered by the nucleoprotein N, which is highly expressed within infected cells (29–31). Covering the nucleocapsid, yet beneath the envelope is the matrix protein M, which has been reported to travel to the nucleus of infected cells during the replication cycle of hRSV to inhibit the transcription of host genes and was recently described to interact with actin within infected cells, likely contributing to the transport of virion components into budding virions (30, 32, 33). Importantly, the virion envelope is covered on its surface by the attachment glycoprotein G, which may be dispensable for infection in some cells (34–36), the fusion F glycoprotein which is a type-I integral membrane protein that binds nucleolin for cell infection (37, 38), and the transmembrane protein SH, which forms a viroporin that transports cationic ions (39, 40). Importantly, to date, there is accumulating data that describes the molecular interactions between hRSV structural components, which has allowed establishing an overall comprehensive scenario of how the virus' components are coordinately assembled within infected cells to favor its replication and exit (30). Finally, the non-structural (NS) genes NS1 and NS2 that are at the foremost 3′ of the viral genome, are solely expressed within infected cells (not contained within the virion), and are known to negatively modulate the cellular antiviral interferon type-I response early after infection (Figure 1) (41, 42). Importantly, several host factors that modulate the replication cycle of hRSV, such as factors involved in the regulation of host transcription, innate immune responses, regulation of the cytoskeleton, membrane remodeling, and cellular trafficking have been identified and confirmed. These factors could eventually be overexpressed or silenced in host cells to reduce infection or hamper virus replication during infection to avoid pathology (43–45). Although many of the abovementioned hRSV proteins have been studied individually *in vitro*, only few of them have been assessed within immune cells or more specifically DCs, which if performed could eventually reveal relevant immune-evasion or immune-modulation properties for hRSV-encoded viral factors and help identify key viral and host factors that modulate the virus' replication cycle in these cells.

## hRSV Infects Dendritic Cells

Dendritic cells (DCs) are immune cells that play vital roles in initiating and regulating antigen-specific immune responses against foreign and self-antigens in the organism (46–48). DCs are strategically located both, at peripheral sites and internal organs in such a way to sense and capture both, foreign and self-proteins. If the captured protein is immunogenic or associated with activating molecules, DCs undergo phenotypic transformations, and migrate to lymph nodes (LNs) to present protein-derived peptides to antigen-specific CD8^+^ and CD4^+^ T cells in MHC-I and MHC-II molecules, respectively (49–52). Importantly, DCs express a battery of molecular sensors that detect pathogen-associated molecular patterns (PAMPs), which leads in most cases to transcriptional and phenotypical changes in these cells in a process known as DC maturation (46, 53, 54). In turn, this process will lead to the activation and modulation of other immune cells that can help resolve infection (49, 50, 55, 56). If DCs capture virus components DCs, these cells will ideally encounter, activate and differentiate virus-specific CD4^+^ T cells into helper cells (Th) that support the production of antiviral antibodies by B cells, as well as promote the generation of cytotoxic T cells (CTLs) that eliminate virus-infected cells (57–59). Given the crucial role of DCs in initiating antigen-specific adaptive antiviral immune responses, mainly through the activation and differentiation of T cells, such as CD4^+^ T helper cells, numerous viruses and other pathogens have evolved molecular determinants and mechanisms to interfere with the function of DCs, in such a way to impair the establishment of an effective antiviral immune response (60–67).

Noteworthy, hRSV infects DCs *in vitro* and is known to interfere with their functions, even though DCs seem not to be an optimal viral substrate for this virus. Indeed, many *in vitro* studies report relatively low virus yields from hRSV-infected DCs, even at multiplicity of infection (MOI) values that generally lead to complete infection of epithelial cell cultures (MOI >3) (66, 68–71). This phenomenon is suggestive of abortive hRSV infection in a significant proportion of DCs (66, 68, 69, 71, 72). Thus, it seems that hRSV likely infects DCs as a strategy to target a pivotal immune component to indirectly favor its infectious process in the host, namely the infection of epithelial lung cells that yield high amounts of infective virions, which will expand the magnitude of the infection within the individual and promote its dissemination onto others. Interestingly, hRSV may reach other tissues besides the airways during infection, such as the central nervous system (CNS) (73, 74).

**Figure 2:**
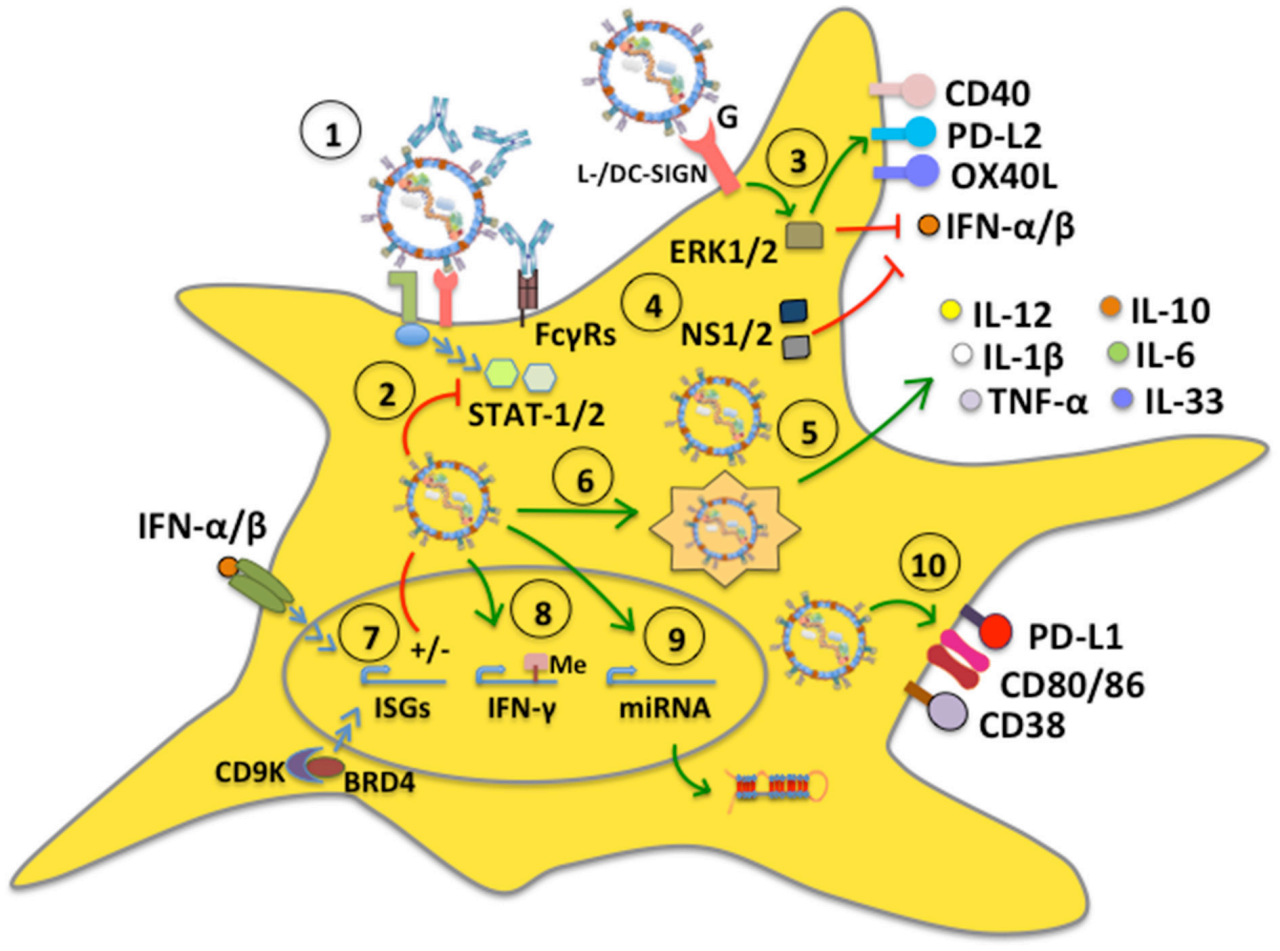
hRSV modulates dendritic cell function. (1) DC infection with hRSV can occur even in the presence of antibodies bound to the virus (opsonized virus), which enter DCs through Fcγ receptors (FcγRs). (2) hRSV is capable of inhibiting antiviral signaling pathways mediated by STAT-1 and STAT-2, likely through its NS proteins. (3) The G glycoprotein signals through L-/DC-SIGN and phosphorylates ERK1/2, which translates into the upregulation of surface expression of CD40, OX40L, and PD-L2, whereas it downregulates IFN-α secretion. (4) The hRSV NS1 and NS2 proteins interfere with type-I interferon secretion. (5) hRSV induces the secretion of proinflammatory cytokines by DCs. Some mDC subsets (BDCA-1^+^ and BDCA-3^+^) secrete IL-10. (6) hRSV induces autophagy and is processed by the autophagosome leading to cytokine release and lung inflammation. (7) hRSV differentially modulates the expression of interferon-stimulated genes (ISGs), through IFN-dependent and independent pathways. (8) hRSV induces the activity of demethylases to modulate gene expression, such as IFN-γ, preventing an antiviral response. (9) hRSV upregulates the expression of specific host microRNAs. (10) hRSV stimulates the expression of CD80 and CD86. Additionally, the virus upregulates PD-L1 and CD38 expression on the DC surface to modulate inflammation in the lungs.

Although cell surface receptors that lead to hRSV cell infection have been identified, such as cellular heparan sulfate glycosaminoglycans that act as attachment factors for the hRSV G glycoprotein (75, 76), as well as nucleolin (37) and ICAM1 (77) as ligands for the F fusion protein, the exact mechanism by which hRSV enters DCs has not been corroborated and could eventually be different compared to that observed in other cells, such as epithelial cells (78). Noteworthy, opsonized hRSV particles (hRSV covered with virus-specific antibodies), which is known to hamper virus-infection of epithelial cells, were recently shown to be nevertheless capable of infecting DCs and interfere with their function, such as activating T cells (Figure 2). Importantly, this process was shown to be mediated by Fcγ receptors (FcγRs) expressed on the surface of DCs (79). Because opsonized hRSV particles retained the same ability as free hRSV to interfere with DC activation of T cells, this process would favor impaired DC function in time despite the individual having anti-hRSV antibodies. Thus, hindered DC function by hRSV would ensue during each exposure to the virus, likely hampering the capacity of the host to mount an effective response against this virus.

## hRSV-Dendritic Cell Interaction

Growing amounts of studies have focused on the relationship between hRSV and DCs increasing our knowledge on the outcome of this interaction. While some reports indicate that DCs infected with hRSV can sense viral components, which can lead to somewhat activating signaling pathways within these cells, other reports indicate that hRSV determinants interfere with antiviral signaling pathways within DCs, such as those related to interferon type-I responses, which is mediated by STAT-1 and STAT-2 (80). Importantly, the activation or inhibition of distinct intracellular signaling pathways within DCs by hRSV generally leads to DC outcomes that are overall poorly activating for T cells, with hRSV-infected DCs displaying poor- or only partial-maturation phenotypes both, in human and murine DCs (58, 59, 66, 68, 69, 72, 81).

NS1 and NS2 have been reported as two hRSV factors that are directly involved in inhibiting the maturation of human DCs and impairing the secretion of type-I IFNs by myeloid DCs (mDCs), which can enhance the differentiation of CD4^+^ T cells into Th2-phenotypes and promote the generation of Th2-polarized anti-hRSV immune responses in the host. Consequently, these immune responses can be detrimental to the host, as they promote exacerbated inflammation in the lungs (80, 82). Another hRSV-encoded determinant that has been reported to alter the maturation of DCs directly is the surface glycoprotein G, which was described to trigger ERK1 and ERK2 phosphorylation within these cells, mainly through DC- and L-SIGN molecules on the DC surface. Neutralization of DC- and L-SIGN induced significant secretion of IFN-α, MIP-1α, and MIP-1β in plasmacytoid DCs (pDCs) inoculated with hRSV, suggesting that this virus alters DC maturation through this signaling pathway thanks to this glycoprotein (Figure 2) (83). Such intracellular signaling events induced by the hRSV G glycoprotein in these cells may explain why mice immunized with a recombinant vaccinia virus (rVV) expressing hRSV G and subsequently challenged with hRSV displayed lung inflammatory DCs that expressed increased levels of the programmed cell death 1 ligand 2 (PD-L2), as well as low CD40 and OX40 ligand (OX40L), when compared to mice inoculated with a rVV expressing the hRSV F fusion protein, which were also challenged with hRSV. Noteworthy, the expression or not of these co-stimulatory molecules on the DC surface was shown to have profound effects over T cell activation, suggesting that the hRSV G glycoprotein has some important immune-modulatory properties, possibly mediated through DCs (84).

Other studies have found that hRSV infection promotes DC maturation and the secretion of pro-inflammatory cytokines by these cells, either directly or through the infection of other cells. For instance, primary human DCs characterized as mDC1, mDC2 or pDC were found to upregulate phenotypic markers associated to maturation after hRSV inoculation, which was dependent on divalent cations suggesting the participation of C-type lectin receptors in this process (71). Other human DC subsets studied with hRSV have been BDCA-1^+^ and BDCA-3^+^ mDCs obtained from peripheral blood. Similar to other DC subsets, these cells were susceptible to infection with hRSV, and while they expressed increased amounts of CD80 and CD86 in response to this virus as compared to non-infected cells, they also expressed the inhibitory costimulatory receptor PD-L1 and secreted IL-10. Furthermore, hRSV-infected BDCA-1^+^ mDCs produced pro-inflammatory cytokines and chemokines, namely IL-1β, IL-6, IL-12, MIP-1α, and TNF-α and displayed a reduced capacity to stimulate T cells (85). Hence, hRSV can produce significant changes in DCs once infected, namely by modulating the expression of T cell-activating molecules on their surface. etSuch modulation was accompanied by the expression of inhibitory receptors and the secretion of numerous immune-modulatory cytokines, mostly inflammatory.

Another study reported that depending on the hRSV strain used; human DCs can respond differentially to this virus by secreting different kinds of type-I and type-III IFNs, and transcribe distinct interferon-stimulated genes (ISGs). Although both serotypes of hRSV A and B induced the expressing of IFN-β, IFN-α1, IFN-α8, and IFN-λ1-3, only the serotype A2 induced IFN-α2, -α14, and -α21 (86). Type-I IFN-dependent activation of ISGs during an hRSV infection was shown to be modulated by the virus' ability to downregulate suppressor of cytokine signaling (SOCS1 and SOCS3) through its RSV G protein, in turn affecting IFN-β and ISG15 expression (87). Moreover, during hRSV infection, airway epithelial cells activate cyclin-dependent kinase 9 (CDK9) and associates with bromodomain 4 (BRD4) to activate IRF3-dependent IFN-stimulated genes, independent of IFN-signaling. Altogether, these processes contribute to increased RSV-induced airway inflammation and disease (88, 89). Although these findings may have important implications over disease severity and the outcome of the host's immune response, as well as the modulation of immunity, the implications of different hRSV serotypes in clinical infections and *in vitro* studies are somewhat seldom assessed.

IL-33 is a key cytokine involved in Th2 immune responses and inflammatory airway diseases and is usually secreted in high amounts by epithelial cells in this tissue (90, 91). Interestingly, hRSV-infected DCs within the lungs of hRSV-infected animals have been reported to have elevated levels of IL-33 mRNA and were suggested to be a relevant source of IL-33 in the lungs of hRSV-infected mice (92). Noteworthy, blocking TLR3 or TLR7 signaling with antagonists significantly reduced the levels of IL-33 mRNA produced by DCs, suggesting that IL-33 expression in these cells upon hRSV infection is TLR-dependent (92). Interestingly, a study on the identification of enzymes that alter the methylation status of the host DNA suggests that the profile of cytokines secreted by DCs in response to hRSV may be driven by specific demethylases induced by infection with this virus (Figure 2). In a study by Ptaschinski and colleagues, it was shown that hRSV upregulates the expression of Kdm5b/Jarid1b H3K4 demethylase in response to *in vitro* hRSV infection of DCs and that inhibiting this factor with siRNA led to a 10-fold increase in IFN-β production, as well as other cytokines. Furthermore, mice that had Kdm5b specifically deleted in DCs showed higher production of IFN-γ and reduced IL-4 and IL-5 secretion after hRSV infection, as well as lesser lung inflammatory mucus production in this tissue. Some of these findings were mirrored in human DCs treated with an inhibitor of KDM5B suggesting that this factor, which is induced by hRSV can directly inhibit the expression of type-I IFN and other cytokines within infected DCs, likely favoring hRSV replication and virus-induced lung disease (93). This finding calls for further studies assessing the roles of such DNA-modification enzymes in host cells and how they are modulated by hRSV infection, potentially unveiling novel antiviral strategies.

Studies that have assessed the role of autophagy in hRSV-infected DCs have found that this process is involved in driving the production of cytokines that lead to lung inflammation (Figure 2). Indeed, inhibition of autophagy with inhibitors such as siRNA, or experiments with Beclin^+/−^ mouse-derived DCs, or exposing Beclin^+/−^ mice to hRSV significantly reduced the production of cytokines by CD4^+^ T cells. In these cases, hRSV-infected lungs displayed increased amounts of mucus secretion, and cellular infiltrates, unveiling important roles for autophagy in DCs in response to hRSV infection (94, 95). Additionally, Beclin-1^+/−^ DCs were shown to express reduced amounts of MHC class II molecules on their surface and were less effective at stimulating the production of IFN-γ and IL-17 in co-cultures with CD4^+^ T cells, as compared to controls; furthermore, they promoted the secretion of Th2-cytokines by these T cells. On the other hand, transfer of hRSV-infected Beclin-1^+/−^ DCs into the airways of wild-type mice elicited lung disease accompanied with the production of significant amounts of Th2 cytokines upon later challenge with hRSV (94, 95). Notably, a recent study found that hRSV induces Sirtuin-1 (SIRT1) expression in DCs, which is a NAD(+)-dependent deacetylase that is associated with the induction of autophagy. In this study, it was found that SIRT1 exerts antiviral effects against hRSV *in vitro* and that using an inhibitor of this enzyme, siRNA o analyzing the specific effect of SIRT1 knockout in DCs not only attenuated autophagy in these cells, but these animals manifested exacerbated hRSV-pathology (96).

When searching for particular markers induced by hRSV infection in DCs or cytokines elicited by hRSV-infected DCs, a recent study found that this virus induces CD38 expression in these cells, which is an ectoenzyme that catalyzes the synthesis of cyclic ADPR (cADPR). The expression of this enzyme was found to be dependent on hRSV-induced type-I IFN and inhibitors of CD38 significantly reduced the expression of type-I/III IFNs, suggesting that CD38 is regulated by- and influences IFNs in DCs and thus, modulating this enzyme may be an intriguing target for improving the host's response to hRSV infection and pathology (97).

Despite poor, or relatively low expression of surface markers associated with the potential capacity of DCs to activate or promote the activation of T cells, a common feature that has been repeatedly observed in hRSV-infected DCs is the secretion of cytokines that may promote the differentiation of T cells into phenotypes that are not favorable for the effective resolution of infection, such as IL-6 and IL-10, which lead to Th2 CD4^+^ T cell responses (66, 81). Concomitantly, cytokines such as IL-12 that tend to elicit T cells with phenotypes that are commonly associated with efficient viral clearance, such as Th1 are usually not secreted by hRSV-infected DCs, (66, 68, 69, 98–101). Interestingly, a study reported that the secretion of different cytokine profiles by hRSV-infected human DCs depends on whether these cells originate from neonates or adults. For instance, DCs derived from blood cord samples secrete more TGF-β1 than DCs obtained from adult blood in response to hRSV, suggesting the existence of age-related phenotypes in DCs that may translate into differential responses to hRSV (further discussed below) (102).

Interestingly, a somewhat novel approach that is being undertaken to study the relationship between hRSV and DCs is analyzing the profile of miRNA expression in these cells (Figure 2). A recent study found that DC infection with hRSV elicited the upregulation of a specific miRNA, namely let-7b (103). This study is complemented by another report that found that hRSV infection induces significant expression of three miRNAs, namely hsa-miR-4448, hsa-miR-30a-5p, and hsa-miR-4634 in human DCs (104). Interestingly, this latter study also performed comparative analyses of miRNA profiles between DCs infected with hRSV and a related virus, namely the human metapneumovirus, and found that both viruses induced the expression of elevated levels of hsa-miR-4634. Elucidating the contribution of these miRNAs in DCs in response to hRSV remains to be determined.

## Dendritic Cell Phenotype and Migration Upon hRSV Infection *in vivo*

**Figure 3:**
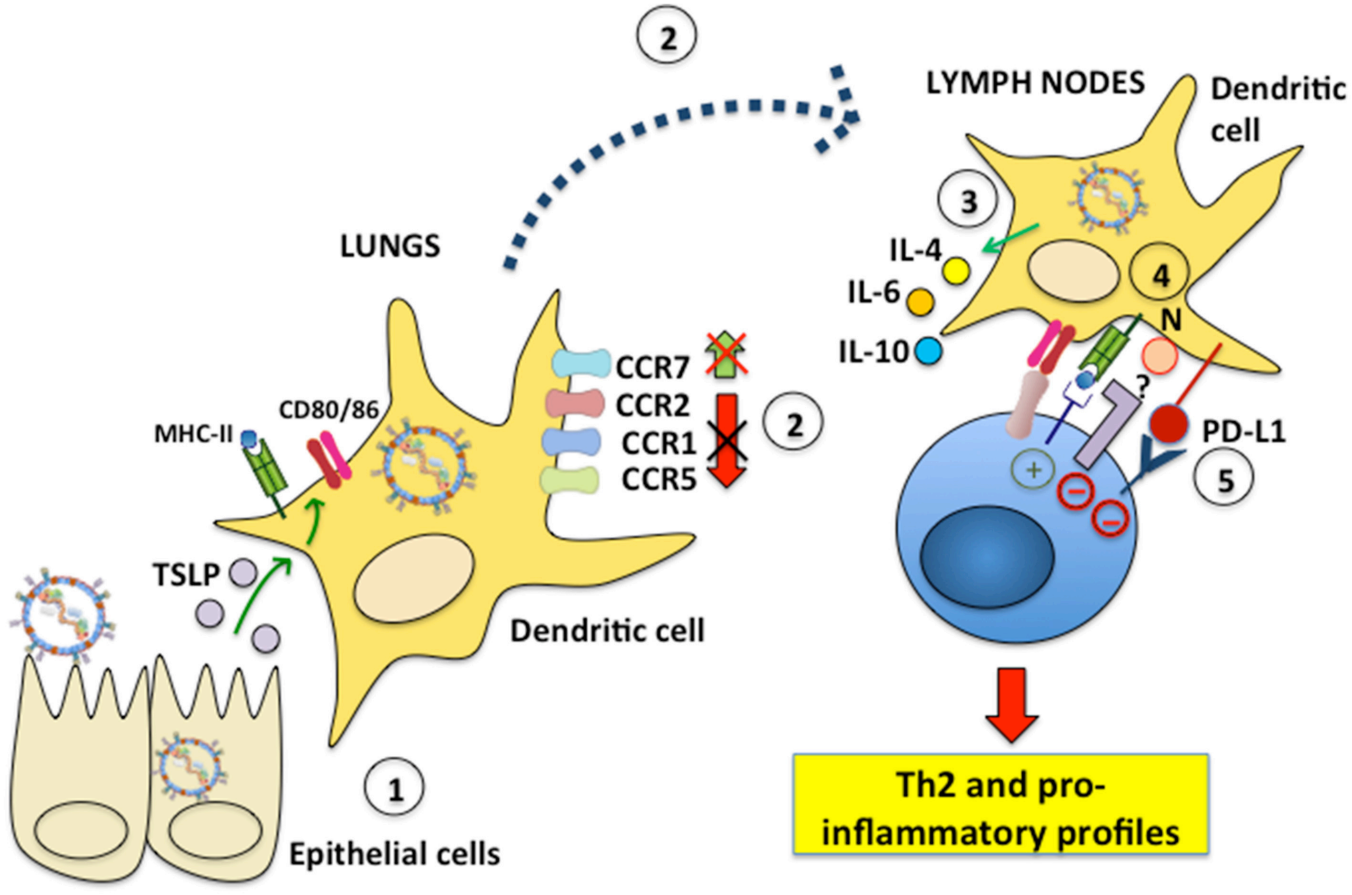
hRSV modulates dendritic cell migration and induces inflammatory profiles in T cells. (1) Epithelial cells infected with hRSV produce TSLP, which elicits MHC-II, CD80, and CD86 expression in lung DCs. (2) hRSV-infected DCs fail to downmodulate the surface expression of the chemokine receptors CCR1, CCR2 and CCR5, which is needed for effective DC migration to lymph nodes. Additionally, CCR7, which favors DC migration to the LNs, is not upregulated on the surface of hRSV-infected DCs. (3) Nevertheless, during infection hRSV-infected DCs migrate to the LNs to interact with T cells. hRSV-infected DCs secrete IL-4, IL-6, and IL-10 and promote the differentiation of CD4^+^ helper T cells toward a Th2 phenotype. (4) The hRSV N protein expressed on the DC surface mediates impaired immunological synapse assembly. The signaling events led by host proteins that interact with N are unknown. (5) hRSV induces PD-L1 expression on the DC surface which signals negatively within inflammatory T cells.

Although the study of DC infection with hRSV *in vitro* has provided valuable insights on the consequences that hRSV infection has over these cells, studying the effects of hRSV over DCs at the site of infection is likely key for understanding the contribution of this interaction to airway disease. They are also important as they help determine if the results obtained *in vitro* mirror what occurs in the respiratory tissue. Interestingly, several studies have addressed the question of how DCs respond to lung infection with hRSV, yet only a few have directly assessed whether the analyzed DCs are actually infected with hRSV, or if the observed effects are driven by viral antigen or other factors in the virus-infected environment. Evidence for the participation of hRSV-infected DCs in the exacerbated inflammatory response to hRSV has been reported by the instillation of hRSV-infected DCs directly into the airways, which produced a pathological Th2-type response in mice (105). Regarding how DCs are infected by hRSV *in vivo*, a study by Ugonna et al. explored in an *in vitro* setting whether cells present in the respiratory tissues may contribute to hRSV access to DCs. Interestingly, by analyzing the interrelationship between DCs and epithelial cells, and their reciprocal infection in co-culture transwell assays they found that macrophages on the apical surface of differentiated epithelia helped hRSV infect DCs in the basal chamber, suggesting that lung macrophages may have a potentially relevant, and previously unknown role in DC infection with hRSV (106). However, this remains to be assessed and demonstrated in *in vivo* settings. Furthermore, other reports have analyzed whether cells that are usually adjacent to DCs in the infected tissues may influence the outcome of DCs. Interestingly, one study found that hRSV-infected rat airway epithelial cells elicited DC activation, increasing MHC-II and CD86 surface expression, as well as enhancing T cell proliferation in mixed lymphocyte reactions. Noteworthy, this activation was dependent on thymic stromal lymphopoietin (TSLP), a pleiotropic cytokine implicated in inflammatory diseases, which was secreted by hRSV-infected airway epithelial cells (Figure 3) (107, 108). On the other hand, airway DCs incubated with inflammatory mediators secreted by hRSV-infected lung epithelial cells was shown to induce their differentiation into functional DCs capable of activating T cells characterized by a type-I IFN antiviral response. Nevertheless, these DCs only had a partial mature phenotype, as they were unable to up-regulate CD80, CD83, CD86, and CCR7, and were unresponsive to TLR triggering, suggesting that the airway epithelium elicits DCs with a somewhat suppressive phenotype, even under inflammatory conditions induced in the lungs after infection with hRSV (109).

Given the existence of diverse types of DCs in the lung tissue, different studies have focused on analyzing the effects of hRSV over distinct subtypes of DCs in the respiratory tissue and their contribution to hRSV-associated lung pathology (110). Early studies on the dynamics of DCs *in vivo* in the lungs of hRSV-infected animals showed that pDCs accumulate in this tissue and secrete type-I IFNs, thus contributing to limit viral replication and the extension of pathology induced by hRSV infection (111, 112). Interestingly, other subsets besides pDCs, such as conventional DCs (cDCs) also accumulate in the lungs of hRSV-infected animals (113, 114). Noteworthy, together with increased accumulation of DCs in the lungs, an increase in the amount of these cells in the associated LNs also occurs, with DCs exhibiting varying phenotypes at this site (114–118). However, in most cases, it is unclear whether the analyzed DCs are infected by hRSV, or if their migration is influenced by other factors within the infected tissue, such as hRSV antigens or cytokines elicited in the infected tissue.

Importantly, differences in the phenotype of DCs obtained from the lungs of hRSV-infected animals have also been assessed based on the age of the individual, by analyzing these cells in the lungs of neonates and adults. One such study found that while CD103+ DCs dominated the response to hRSV in neonates, CD11b^+^ DCs were underrepresented in this group both, in number and function as compared to adult animals. For instance, pDCs from neonate animals display limited type-I IFN responses during hRSV infection, as compared to adult pDCs (119). Noteworthy, the transfer of adult pDCs into neonate animals reduced the Th2-biased immunopathology produced by hRSV which was elicited after a subsequent challenge with hRSV, further evidencing significant differences between DCs obtained from these different age-groups in response to hRSV infection (120). In line, with this notion, another study found that neonatal CD11b^+^ mDCs expressed increased levels of the IL-4 receptor IL-4Rα, as compared to adult DCs and that specifically deleting this cytokine receptor from CD11b^+^ mDCs significantly decreased hRSV-induced immunopathophysiology. Concomitantly, overexpression of IL-4Rα on the surface of CD11b^+^ DCs of adult animals and transferring them into adult mice elicited hRSV-induced immunopathology. Finally, an important finding in this study was also the fact that increased IL-4Rα expression in DCs was associated with reduced maturation of DCs during hRSV infection (121). Interestingly, another study found that age-dependent DC responses against hRSV could be modified through the use of TLR agonists, such as agonists for TLR4 or TLR9 at the time of infection. By using such agonists, a significant change in the response of hRSV-specific CD8^+^ T cells could be observed, evidenced as a shift in the immunodominance of the antigens to which these T cells responded when activated by neonate DCs. The shifted response found resembled more that was observed in adults, which is associated with less severe disease (122). Overall, the findings outlined above suggest particular and distinctive features between lung DCs from neonates and adults after hRSV infection, at least in the mouse model, and could be considered in the future for potential therapeutic and prophylaxis approaches in neonates and adults.

Another area of intense research regarding the interaction between hRSV and DCs is the migration of these cells, as hRSV lung infection may result in alterations on the of migration pattern of different subsets of DCs from the lungs to LNs. Interestingly, an *in vitro* study found that human monocyte-derived DCs infected with hRSV failed to downregulate CCR1, CCR2, and CCR5 from their surface, which is required for DCs to effectively migration to LNs. Indeed, these infected DCs migrated significantly less in chemokine gradients in *in vitro* assays. Furthermore, hRSV-infected DCs failed to upregulate CCR7 on their surface, which is known to promote the migration of antigen-exposed DCs to LNs for presenting antigens to T cells (123). Nevertheless, these findings need to be corroborated in hRSV-infected individuals. Even though cDCs accumulate in the lungs of hRSV-infected animals as mentioned above (113, 114), it has been reported that monocyte DC precursors are depleted during infection from this tissue. Importantly, this phenomenon has been suggested to favor opportunistic lung infections by pathogens, such as bacteria (116). Notably, two major subsets of lung tissue cDCs have been shown to transport hRSV RNA to the LNs, namely CD103^+^/CD11b^low^/CD11c^+^ and CD103^−^/CD11b^high^/CD11c^+^ cDCs and present hRSV antigens to T cells on MHC-I and MHC-II molecules (118). Interestingly, a study that was mentioned in the section above which analyzed the effects of TLR agonists over DCs infected with hRSV showed that the treatment with these molecules increased the numbers of CD11b^+^ and CD103^+^ DCs migrating from the lungs to draining LNs in neonates, likely supporting an improved antiviral response thanks to DCs with adult-like phenotypes migrating to this site for optimal T cell activation (122).

Although some studies suggest a positive role for lung cDCs during hRSV infection, other reports indicate that these cells may play detrimental functions for the host during hRSV infection (111, 113, 115). These effects have been evidenced, for example by blocking the chemokine CCL20 in hRSV-infected animals or knocking-out its associated receptor (CCR6), which significantly reduced the presence of cDCs in the airway tissue without affecting pDCs. These scenarios overall translated into improved outcomes of hRSV infection, suggesting that a balance between pDCs and cDCs in the lungs is likely associated with hRSV-induced pathology (114, 124). The finding supports this notion that depletion of pDCs from the lungs of animals significantly increases pulmonary disease after a challenge with hRSV (112). Concomitantly, activation of pDCs in the lungs of hRSV-infected animals was shown to limit the replication of hRSV in the airways and decrease hRSV-associated pathology (114). Thus, pDCs, as well as cDCs in the airways, are considered to interplay limiting hRSV replication and regulating inflammation (111, 112, 114). However, whether the findings described above in the mouse model hold in humans remains to be determined. Interestingly, some observations performed in animal models have been mirrored in patients, such as individuals experiencing hRSV bronchiolitis having significantly higher numbers of cDCs than pDCs in the blood, suggesting an imbalance in the proportion of DC subtypes in children with bronchiolitis, as compared to healthy individuals after hRSV infection (125).

## Molecular Mechanisms Involved in the Reduced Capacity of hRSV-infected DCs to Activate T Cells

A substantial effect of hRSV over DC function is its ability to reduce the capacity of hRSV-infected DCs to effectively activate CD4^+^ and CD8^+^ T cells (Figure 3). Although this phenomenon has been reported *in vitro* and is not necessarily echoed *in vivo* in humans or animal models, a relationship between the *in vitro* observations and potential *in vivo* effects likely exists in terms of non-optimal T cell activation taking place, as a result of DC infection with hRSV (66, 68, 72, 126). Given that the reported phenotype of hRSV-infected DCs is generally associated to weak or incomplete maturation, it is somewhat expected that the activation of T cells by infected DCs will not be optimal and may lead to less potent, or inadequately differentiated or polarized hRSV-specific T cells. Interestingly, some studies have identified particular hRSV factors that are involved in hampering the capacity of DCs to activate T cells. For instance, a report published in 2008 that studied the interaction between DCs and hRSV suggested that hRSV factors driving poor T cell activation by hRSV-infected DCs were membrane-bound and interfered with the establishment of the immunological synapse (IS) between T cells and hRSV-infected DCs *in vitro*, which is essential for productive T cell activation (49, 66). This notion was reinforced by the fact that supernatants from hRSV-infected DCs enhanced the activation of T cells in the presence of plate-bound activating anti-CD3 and anti-CD28 antibodies (66). However, another study suggested that soluble factors secreted by hRSV-infected DCs were involved in impaired T cell activation by hRSV-infected DCs (68, 99). It is possible thus that both, membrane-bound and soluble factors secreted on to the extracellular media, or at the DC-T cell immunological synapse negatively modulate the activation of T cells, given that the is highly susceptible to modulation by factors of both natures (49).

A membrane-bound hRSV factor that has been reported to mediate negative effects over T cells is the hRSV F fusion protein, which expressed on the surface of epithelial cells was shown to inhibit T cell activation *in vitro* (127). However, the effect of the hRSV F protein has not been assessed in the context of DC-T cell interactions. Still, a study that assessed the role of the hRSV N nucleoprotein in mediating detrimental effects over the DC-T cell interaction found that this protein was present on the surface of hRSV-infected DCs and could directly mediate the inhibition of T cell activation (128). An interesting finding in this study was the fact that the hRSV N protein was shown to be able to interfere with the establishment of productive immunological synapses between T cells and cognate ligand mounted on lipid bilayers (128). Importantly, the identification of the hRSV N protein on the surface of infected cells had not been previously reported for this virus. Another hRSV factor known to hamper the capacity of DCs to activate T cells is NS1, which has been found to negatively modulate the capacity of human DCs to activate both, CD4^+^ and CD8^+^ T cells (129). Additionally, NS1 has also been reported to favor the differentiation of DCs toward phenotypes that promote the activation of CD4^+^ T cells that secrete IL-4, yet by a mechanism that is independent of its capacity to modulate IFN-I signaling (129).

Again, depending on whether DCs inoculated with hRSV originate from neonate or adult animals, a study by Thornburg et al. reported differences in the capacity of such hRSV-infected DCs to activate autologous T cells, with DCs from adult mice eliciting IFN-γ, TNF-α, and IL-12 secretion in co-cultures and neonate DCs (from blood cords) eliciting IL-1β, IL-4, IL-6, and IL-17 release (102). Furthermore, neonatal CD103^+^ DCs have been shown to promote the proliferation of T cells differently, as compared to adult CD103^+^ DCs, namely by eliciting the expansion of T cells against distinct antigens, defining different hRSV antigenic hierarchal profiles. The differences observed with DCs from animals of different ages in this and other studies, suggest that neonatal DCs overall display limited T cell co-stimulatory properties when compared to adult DCs, which could eventually relate to infants being more susceptible to severe disease than adults after hRSV infection (119). Another report found two phenotypically and functionally distinct populations of CD103^+^ DCs in the lungs of neonatal mice following hRSV infection, and that those that were CD103^lo^ were functionally limited at activating hRSV-specific T cells, while those that were CD103^hi^ were capable of potently activating T cells (130). Whether such differences mirror the adaptive immune responses to hRSV *in vivo* in both adults and infant humans, remains to be determined.

Although several studies have reported impaired T cell activation by hRSV-infected DCs *in vitro, in vivo* studies reveal that hRSV-specific T cells are expanded in the organism after infection, yet they generally display pro-inflammatory phenotypes that likely contribute, or are the root of exacerbated lung damage during hRSV infection (131–133). Thus, reduced activation of T cells *in vitro* seems to translate *in vivo* as the activation of virus-specific T cells with detrimental phenotypes that respond to an hRSV lung infection. Yet, a role for hRSV-infected DCs has also been described in regulating or controlling pathogenic T cells during infection. Analyses of human and murine lung DCs report that these cells express PD-L1 and that this molecule is critical for suppressing the activity of inflammatory T cells (Figure 3). This finding suggests a vital role for the PD-L1/PD-1 axis in DC-T cell interactions for limiting the inflammatory response of T cells to hRSV (134). Nevertheless, the results of this study contrast with those of another report that found that hRSV inhibited the capacity of pDCs to produce a regulatory T cell response to inhaled antigens, eliciting an alteration in their immunotolerogenic potential (135).

Because of the key role of DCs in mounting and regulating immune responses to viruses such as hRSV, novel vaccines are needed to strategically seek and target these cells in a specific manner. A recently described approach that directly involves DCs consists on a DNA vaccine encoding the ectodomain of the hRSV F protein fused to a single-chain variable fragment F (scFv) that directly targets the viral antigen to DEC205 on the DC surface. This viral protein is then phagocytosis by DCs through this receptor and processed for antigen presentation to T cells. Interestingly, this strategy has been reported to elicit high levels of anti-hRSV antibodies with neutralizing capacity and induce F-specific CD8^+^ T cells that elicit a Th1 response in mice (136).

## Concluding Remarks

Over the last years, new studies have revealed novel features of the DC-hRSV interaction, providing unanticipated outcomes in DCs after infection with this virus and helping identify different host and viral factors that participate in these processes. Because DCs play pivotal roles in initiating and regulating antigen-specific immune responses to infections, it seems relevant that particular focus should be given to these cells both, before and after interacting with hRSV. Indeed, these cells are needed for establishing an effective antiviral immune response in the lungs to promote viral clearance, while altogether avoid exacerbated inflammation of the airways. The fact that hRSV can repeatedly reinfect the host without the need of varying its antigens calls for special attention to the steps that determine the founding events of the host antiviral response, in such a way to train the immune system to withstand the negative immune-modulatory properties of this virus or counteract its potent Th-skewing effects. In both cases, a protective immune response elicited against hRSV, such as one that could be induced by a vaccine, should be strong enough to bear subsequent viral reinfections that will push to revert this outcome and elicit scenarios that are favorable for the virus. Overall, significant efforts should be invested in identifying viral and host factors that hamper hRSV-infected DCs, or bystander DCs in the infected tissue from promoting effective antiviral immune responses against this virus. Importantly, promoting positive hRSV-DC interactions during re-infections, after virus-specific immune components have already been polarized toward detrimental phenotypes by hRSV may be more complicated than promoting an effective immune response before primary infection. Indeed, shifting a pre-existing antigen-specific immune profile has proven somewhat challenging in the context of other diseases, such as cancer and autoimmunity, although the antigens involved in these pathologies are seldom known, which is not the case for hRSV.

Finally, some questions that remain open regarding the roles of DCs in hRSV infection are: How can we enable hRSV-infected DCs to elicit effective antiviral immune responses during primary infection and re-infections against this virus? Are there hRSV factors, or hRSV-induced factors elicited during reinfections that revert, through their effects over DCs, otherwise effective primary anti-hRSV immune responses? Do the different circulating hRSV A and B genotypes affect the outcomes of hRSV-infected DCs equally? What are the roles of hRSV-infected and non-infected DCs in the lungs of hRSV-infected individuals? Do the findings reported in the mouse model hold in humans? Hopefully, answers to these and many other questions regarding DCs and their interaction with hRSV will provide novel insights that will help limit the burden and mortality associated with the epidemiology of this important respiratory virus.

## Author Contributions

PG and SB wrote, revised, and edited the article and figures. ET wrote and revised the article and drew the figures.

### Conflict of Interest Statement

The authors declare that the research was conducted in the absence of any commercial or financial relationships that could be construed as a potential conflict of interest.

## Abbreviations

hRSV: Human respiratory syncytial virus
DCs: dendritic cells
IL, interleukin, LNs: lymph nodes.
